# The Interplay between Fe_3_O_4_ Superparamagnetic Nanoparticles, Sodium Butyrate, and Folic Acid for Intracellular Transport

**DOI:** 10.3390/ijms21228473

**Published:** 2020-11-11

**Authors:** Maria Teresa Cambria, Giusy Villaggio, Samuele Laudani, Luca Pulvirenti, Concetta Federico, Salvatore Saccone, Guglielmo Guido Condorelli, Fulvia Sinatra

**Affiliations:** 1 Dipartimento di Scienze Biomediche e Biotecnologiche, Università di Catania, 95125 Catania, Italy; giusyvillaggio@gmail.com (G.V.); s.laudani91@hotmail.it (S.L.); sinatra@unict.it (F.S.); 2 Dipartimento di Scienze Chimiche, Università di Catania, 95125 Catania, Italy; luca.pulvirenti@phd.unict.it; 3 Dipartimento di Scienze Geologiche, Biologiche e Ambientali, Università di Catania, 95125 Catania, Italy; federico@unict.it (C.F.); saccosal@unict.it (S.S.); 4 Consorzio Interuniversitario di Scienza e Tecnologia dei Materiali (INSTM) UdR di Catania, 95125 Catania, Italy

**Keywords:** magnetic nanoparticles, sodium butyrate, surface functionalization, cellular uptake, folate receptors

## Abstract

Combined treatments which use nanoparticles and drugs could be a synergistic strategy for the treatment of a variety of cancers to overcome drug resistance, low efficacy, and high-dose-induced systemic toxicity. In this study, the effects on human colon adenocarcinoma cells of surface modified Fe_3_O_4_ magnetic nanoparticles (MNPs) in combination with sodium butyrate (NaBu), added as a free formulation, were examined demonstrating that the co-delivery produced a cytotoxic effect on malignant cells. Two different MNP coatings were investigated: a simple polyethylene glycol (PEG) layer and a mixed folic acid (FA) and PEG layer. Our results demonstrated that MNPs with FA (FA-PEG@MNPs) have a better cellular uptake than the ones without FA (PEG@MNPs), probably due to the presence of folate that acts as an activator of folate receptors (FRs) expression. However, in the presence of NaBu, the difference between the two types of MNPs was reduced. These similar behaviors for both MNPs likely occurred because of the differentiation induced by butyrate that increases the uptake of ferromagnetic nanoparticles. Moreover, we observed a strong decrease of cell viability in a NaBu dose-dependent manner. Taking into account these results, the cooperation of multifunctional MNPs with NaBu, taking into consideration the particular cancer-cell properties, can be a valuable tool for future cancer treatment.

## 1. Introduction

Colorectal cancer (CRC) and liver and lung metastasis (CLM) represent the leading causes of death in CRC patients; however, there is no agreed-on approach to treat CLM [1]. One of the major problems of anticancer therapies is the emergence of resistance to chemotherapy drugs (multidrug resistance, MDR). For the treatment of a variety of cancers, the combined use of nanoparticles (NPs) and drugs [2] provides a promising strategy to overcome MDR, as well as increasing drug efficacy and decreasing high-dose-induced systemic toxicity [3,4]. For combined therapies, liposomes are the most used NPs but recently the uses of other classes of NPs (such as polymeric nanoparticles and micelles, carbon based nanostructures and metal and metal oxides NPs [2]) have attracted the interest of many researchers. Among them, the use of organic functionalized superparamagnetic iron oxide nanoparticles (MNPs) has attracted increasing attention [5,6] since they combine the versatility of surface functionalization with the magnetic properties and the non-toxic and biodegradable nature of the Fe_3_O_4_ core [7,8]. Functionalized MNPs have been investigated and, in some cases, are already in use for several biomedical applications such as proteins and cell sorting and manipulation [9,10], cell labelling [11,12], magnetic dialysis [13], magnetically controlled drug delivery [14,15], Magnetic Resonance Imaging (MRI) [16,17,18] and magnetic thermotherapy [19,20,21,22]. For combined therapies with MNPs, butyrate can be used as a potential anticancer agent because it has interesting properties for the reduction of the risk and the prevention of CRC [23]. It is present in fruit and vegetables together with other natural substances such as folate, selenium, vitamin D and other short-chain fatty acids (SCFAs). Butyric acid, which is the principal source of energy for normal colonocytes, can promote growth and proliferation, while in cancerous colon cells it inhibits proliferation and induces differentiation and apoptosis [24,25,26]. For this reason, butyrate can be used in cancer therapy since it has a toxic effect on cancer cells but is beneficial for non-cancerous cells. The anti-cancer effect of butyrate has been demonstrated in cancer cell cultures and animal models of cancer [24,27,28].

The present paper studied the effects on the intracellular uptake and cell vitality of the combined use of MNPs modified with two different coatings, and NaBu used as a free formulation. We also investigated the effects of FA as the targeting ligand because it is one of the best-characterized ligands used for targeting cancer cells [29]. In our previous experiments [30] different cellular uptakes were observed for MNPs with (FA-PEG@MNPs) and without (PEG@MNPs) folic acid. In this paper we correlate folate receptors (FRs) expression in LoVo cells with the presence of folate in MNP coatings and, in turn, with cellular uptake. We then evaluated the effects on FRs expression of the contemporary exposure to NaBu and both types of functionalized MNPs.

Moreover, in order to delineate the influence of butyrate delivery on its anticancer activity, the effects of NaBu as a free formulation or in association with MNPs on the growth of LoVo cells were investigated. We wanted to show that the combined use of MNPs and NaBu is a possible strategy to contrast cancer cells because activated multiple pathways involved in various stages of malignant cells.

## 2. Results and Discussion

### 2.1. MNP Functionalization and Characterization

**Scheme 1:**
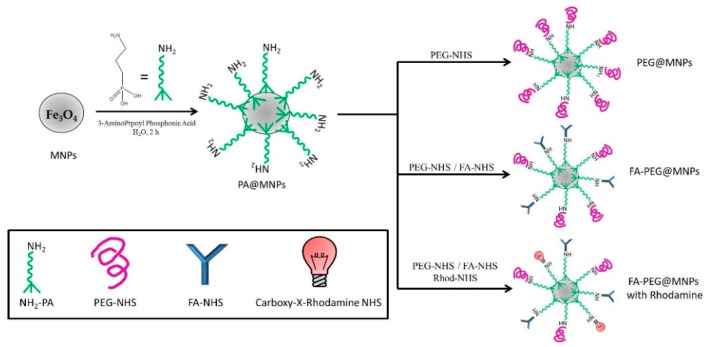
Overall synthetic scheme.

The functionalized MNPs used in this study were obtained adopting a multistep procedure [30,31] based on nanoparticle pre-functionalization with 3-aminophosphonic acids (NH_2_-PA) that is a bifunctional linker able to bind the Fe_3_O_4_ surface through its phosphonic acid group with the amine moiety available for other reactions. N-hydroxysuccinimide (NHS) activated PEG-acetic acid (PEG-NHS) alone or in a mixture (1:1) with NHS-activated FA (FA-NHS) were bonded to NH_2_-PA pre-functionalized MNPs (PA@MNPs) through an amide bond formation in order to obtain PEG@MNPs and FA-PEG@MNPs, respectively. The coating of FA-PEG@MNPs samples used for fluorescence measurements also contained a rhodamine luminescent probe. The overall synthetic route is reported in Scheme 1.

A full characterization of FA-PEG@MNPs with the rhodamine probe was reported in our previous paper [31]. The chemical composition of the synthesized materials was determined by X-ray Photoelectron Spectroscopy (XPS) measurements (Table S1 and Figure S1) and UV-Vis measurements (Figure S2). A transmission electron microscopy (TEM) image of the obtained nanoparticles is also shown (Figure S3). Details of the chemical characterization are discussed in the Supporting Materials.

### 2.2. Cell Viability

**Figure 1:**
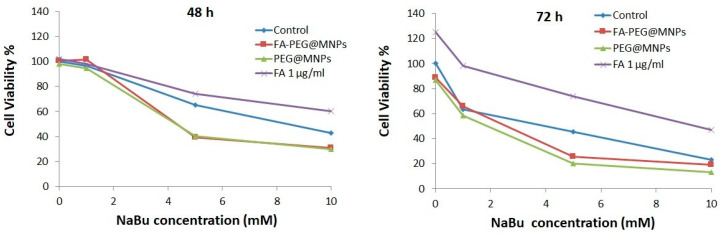
Viability of LoVo cells (MTT assay) incubated for 48 h and 72 h with various NaBu concentrations in the absence (blue line) and the presence of FA (violet line), FA-PEG@MNPs (red line) and PEG@MNPs (green line).

Figure 1 shows the viability of LoVo cells exposed for 48 h and 72 h to various concentrations of NaBu (blue line) as measured by the 3-(4,5-Dimethylthiazol-2-yl)-2,5-Diphenyltetrazolium Bromide (MTT) test. Viability of human colon adenocarcinoma cells in the presence of FA (violet line), or FA-PEG@MNPs (red line) or PEG@(MNPs (green line) together with the free formulations of NaBu are also shown. The effect of NaBu on LoVo cells was very significant as shown by the relevant decrease of the curves observed after 48 h and, even more evident after 72 h. From Figure 1 it is also evident that the reduction in viability is dose-dependent. On the other hand, for all NaBu concentrations, LoVo cells exposed simultaneously to both NaBu and folate (1 μg/mL) showed a greater vitality with respect to the cells treated only with NaBu. Therefore, folic acid protected cancer cells from the cytotoxic effect of NaBu.

NaBu in the presence of FA-PEG@MNPs (red line) and PEG@MNPs (green line) causes a significant cellular viability drop, particularly evident at 5 mM. Both MNPs (in the absence of NaBu) showed a low toxic effect at 72 h, as previously demonstrated [30,32]. This behavior indicated that the combined use of NaBu and MNPs can induce enhanced cytotoxic effects. In particular, the combined effect is most evident at a NaBu concentration of 5 mM, which is close to the physiological concentration. In fact, in normal colonocytes butyrate is present with a concentration gradient ranging from 5 mM in the lumen to 0.5 mM in the basal part of the crypt [33]. From Figure 1, reporting the results obtained after 72 h of exposure, it is also clear that despite the similar profiles, cytotoxicity of the FA-PEG@MNP and the NaBu system was slightly lower than that of PEG@MNPs and the NaBu system, likely because of the FA protective effect.

### 2.3. Cellular Uptake and Localization: SEM/EDX, TEM, and Confocal Fluorescence Microscopy Measurements

Preliminarily, the MNP cellular uptake was studied combining Scanning Electron Microscopy (SEM), Energy Dispersive X-Ray Analysis (EDX), and Confocal Laser Scanning Microscopy (CLSM) measurements. All functionalized MNPs (15 µg/mL) were incubated for 72 h with LoVo cells.

**Figure 2:**
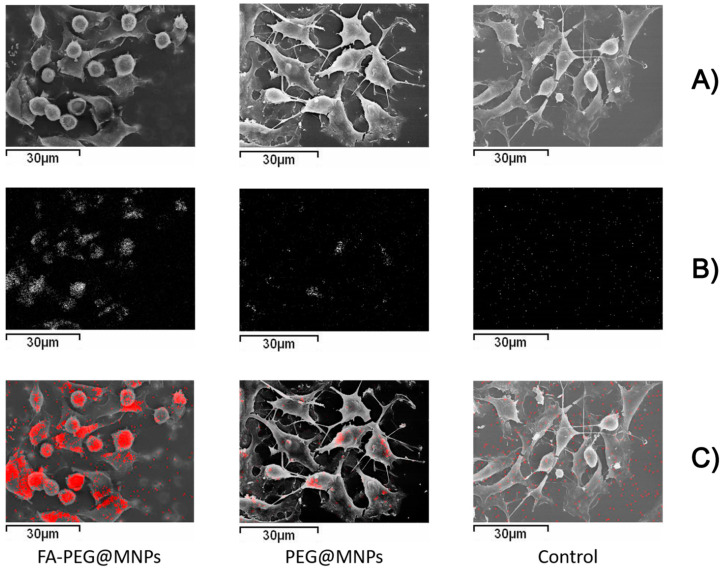
(**A**) SEM images, (**B**) EDX maps of Fe distribution (bright spots), and (**C**) overlay images of SEM and EDX Fe distribution (Fe spots colored red) of samples of untreated cells (control) and cells incubated with either FA-PEG@MNPs or PEG@MNPs.

Figure 2 shows morphological SEM images and EDX maps of Fe distributions of untreated cells (controls), and of cells treated with PEG@MNP and FA-PEG@MNP. SEM images of FA-PEG@MNP treated samples show flattened cells and a higher number of rounded cells compared with the other samples including controls.

EDX maps show that the presence of Fe was evident in the cells treated with FA-PEG@MNP (Figure 2B,C). These results indicate a significant uptake of FA-PEG@MNPs into LoVo cells, whilst it was much less evident for samples treated with MNP without FA. In this case, the amount of Fe inside cells was greatly reduced (Figure 2B,C), in agreement with our previous paper [30].

A more quantitative comparison of the different behavior of MNPs was obtained comparing the average Fe/Au atomic ratio (Figure S4) measured in correspondence to the various cells. The presence of Au was due to the metallization process that produces a homogeneous coating that can be used as internal standard. Typical examples of the region analyzed for determining the Fe/Au ratio in a cell are reported in Figure S4. Results indicated that the presence of FA modified MNPs is almost 10 times higher than that of MNPs without FA. In the presence of 10 mM NaBu with both types of MNPs (PEG@MNPs and FA-PEG @MNPs), the few cells detected with SEM-EDX showed high levels of iron (Figure S5). The observed distribution was not homogeneous but there are accumulations in some areas.

The adopted analytic approach, which combined SEM and EDX measurements, provided a semi-quantitative estimation of cellular uptake without the use of luminescent probes. However, from the EDX measurements it is not possible to determine if MNPs are localized inside the cell or anchored on the cell membrane because of the large EDX sampling depth (about 2 μm [34]). Hence CLSM and TEM measurements were also performed to confirm FA-PEG@MNPs cell uptake and to obtain information on the their localizations in the cells.

**Figure 3:**
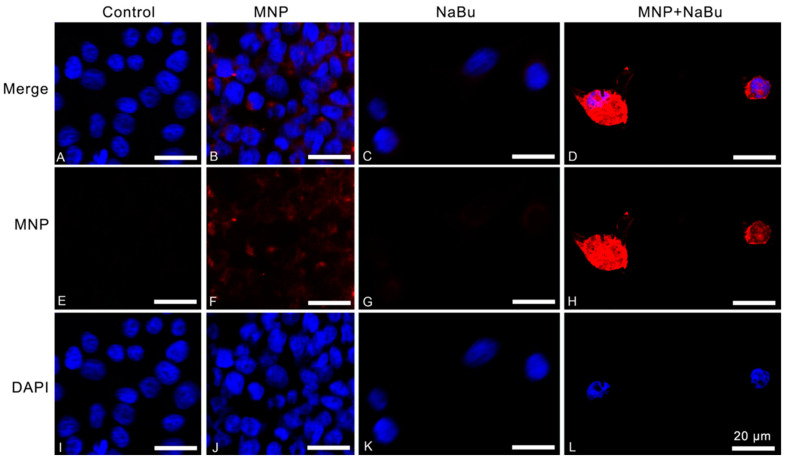
LoVo cells incubated with rhodamine-conjugated FA-PEG@MNPs and/or NaBu. (**A**): LoVo cells not exposed to FA-PEG@MNPs and/or NaBu (negative control). (**B**–**D**): LoVo cells incubated with MNPs, NaBu, and MNPs + NaBu, respectively. Red, and blue signals are due to rhodamine-conjugated FA-PEG@M, and to DAPI stained nuclei, respectively (**E**–**H**): the same images shown in A, B, C, and D, respectively, showing only the red fluorescence due to MNPs. (**I**–**L**): the same images shown in A, B, C, and D, respectively, showing only the blue fluorescence of DAPI staining the cell nuclei. Images were acquired with a Confocal Laser Scanning Microscopy using 405 and 555 nm wavelength lasers to detect the cell nucleus and the FA-PEG@MNPs, respectively. Scale bar: 20 μm.

In order to allow fluorescence monitoring, carboxy-X-rhodamine was added to the FA-PEG@MNP shell (Scheme I). Confocal images show a fluorescent signal with a prevalent localization in the cytoplasm for FA-PEG@MNPs treated cells, whilst no signal was observed in the control (Figure 3). These results are in agreement with our previous study.

**Figure 4:**
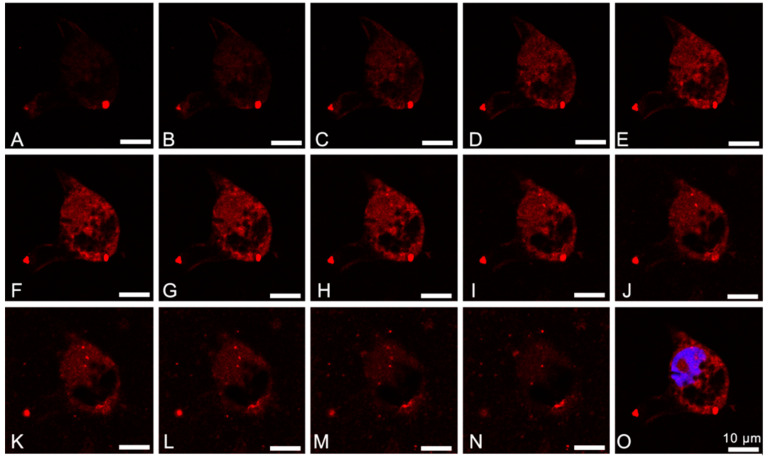
(**A**–**N**): Serial sections with 0.30 µm/section of a LoVo cell co-incubated with FA-PEG@MNP conjugated with rhodamine, and with NaBu. (**O**): Panel corresponding to the central serial section H with the visualization of the nucleus (stained in blue with DAPI). Scale bar: 10 μm.

In the presence of both NaBu and MNPs, the evident fluorescent signal indicated that also in this case MNPs were inside LoVo cells (Figure 3). Moreover, CLSM imagery at different focal planes showed that MNPs were also localized in the nucleus (Figure 4) suggesting that the decrease in cell viability in the presence of NaBu and MNPs was due to growth inhibition and apoptosis.

**Figure 5:**
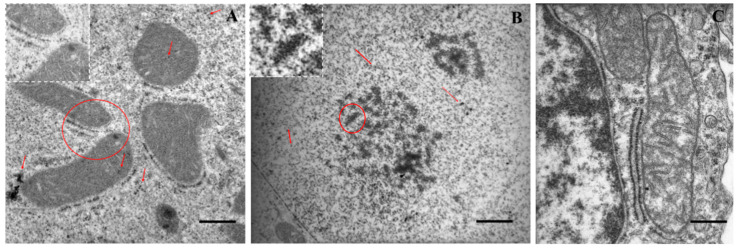
TEM micrographs of (**A**,**B**) LoVo cellular uptake of FA-PEG@MNP after 72 h of incubation, (**C**) control cells. A group of mitochondria (A) and the surrounding cytoplasm contain MNPs (red arrows). In the nucleus FA-PEG@MNP spreads between chromatin (red arrows). The magnified images (white dotted frame) of the area indicated by red circles. Scale bar: A = 0.6 µm, inset: 1.4×; B = 0.7 µm, inset 2.5×; C = 0.6 µm.

The cellular localization of MNPs was also studied through TEM observations. The nanoparticles, after crossing the plasmatic membrane, diffused into the cytoplasm either as monodispersed MNPs or as aggregates. The structure and function of the cytoplasmic organelles, particularly mitochondria, was thus altered to a different extent by the presence of more or less MNPs (TEM micrographs in Figure S6). In Figure 5 the electron transmission micrographs show FA-PEG@MNPs in the mitochondria (Figure 5A) and nucleus (Figure 5B) and an untreated cell (Figure 5C). In the mitochondrial matrix (Figure 5A and inset) MNPs are visible as small spots or externally bounded to ribosomes of rough reticulum. The functionalized nanoparticles in the nucleus were dispersed between the filamentous chromatin (Figure 5B). In particular, where the chromatin is more condensed, as in the nucleolar area, they appear concentrated and distinctly observable thanks to their higher electron density (inset in Figure 5B).

TEM and laser scanning confocal microscopy imagery suggested that butyrate-transcriptional activation in combination with MNPs is indeed able to increase programmed cell death, probably through a nuclear and mitochondrial presence of ferromagnetic nanoparticles. CLSM analysis was also performed to evaluate the presence of the folate receptor in LoVo cells and the effects of NaBu and folate on FRs expression. FRs, known as the high-affinity membrane proteins for folate, are known to be overexpressed in tumor tissue [29].

**Figure 6:**
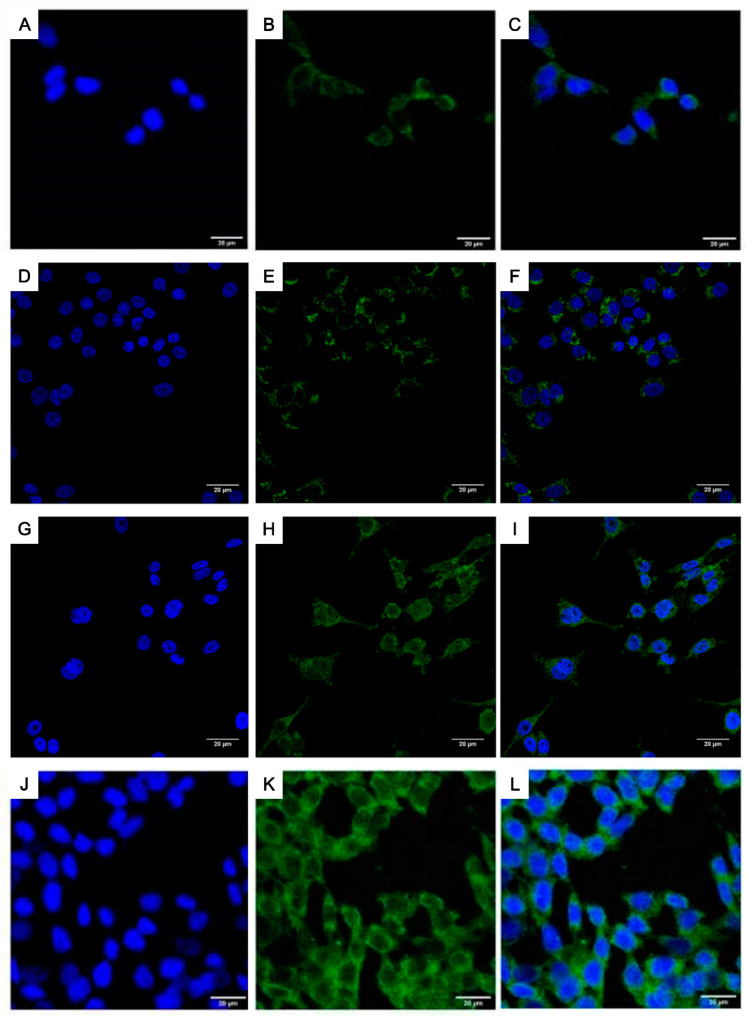
Folate receptors (FRs) analysis (CLSM) in LoVo cells incubated for 48 h (**A**–**C**) alone (control) and with (**D**–**F**) PEG@MNP, (**G**–**I**) FA-PEG@MNP and (**J**–**L**) FA used as a free formulation (1 μg/mL). In A, D, G and J nuclei are stained with DAPI (blue); B, E, H and K green florescence of FRs; in C, F, I, and L a merge was made. Scale bar 20 µm.

Figure 6 compares the presence of FRs in LoVo cells indicated by the green florescence signal after treatment with FA-PEG@MNP and PEG@MNP.

For the cells treated with FA-PEG @MNP (Figure 6G–I), the fluorescence signal was stronger compared to the cells treated with PEG@MNP (Figure 6D–F) and to the control (Figure 6A–C) for the same incubation time. This effect is even more evident (Figure 6J–L) in the case of LoVo cells treated with FA used as a free formulation (1 μg/mL). This behavior indicates that the presence of FA either linked to MNPs or used as a free formulation increases the expression of FRs. The induced FRs overexpression can, in turn, increase the uptake of FA-PEG@MNPs in LoVo cells as shown by EDX and Confocal analyses (Figure 2 and Figure 3). In fact, FRs act as FA-PEG@MNP transporters that determine a greater uptake of FA-PEG@MNP via receptor-mediated endocytosis [35]. In previous studies many roles had been assigned to FRs. FRs, once linked to their ligand, internalize and locate themselves in the nuclear region, acting as transcription factors [36], regulating the expression of some promoter regions. This behavior can account for the observed presence of functionalized MNPs in the nucleus (Figure 4 and Figure 5B). FA-PEG@MNPs diffuse into the nucleus, together with FRs, remaining mainly in the nucleolar zone. FRs have the potential to form macromolecular complexes in which FRs can trigger intracellular signaling. Previous studies strongly suggested that FRα may function not only as a folate transporter, but may also confer signaling and growth advantages to malignant cells [37] and serves as a useful marker for cancer [38].

**Figure 7:**
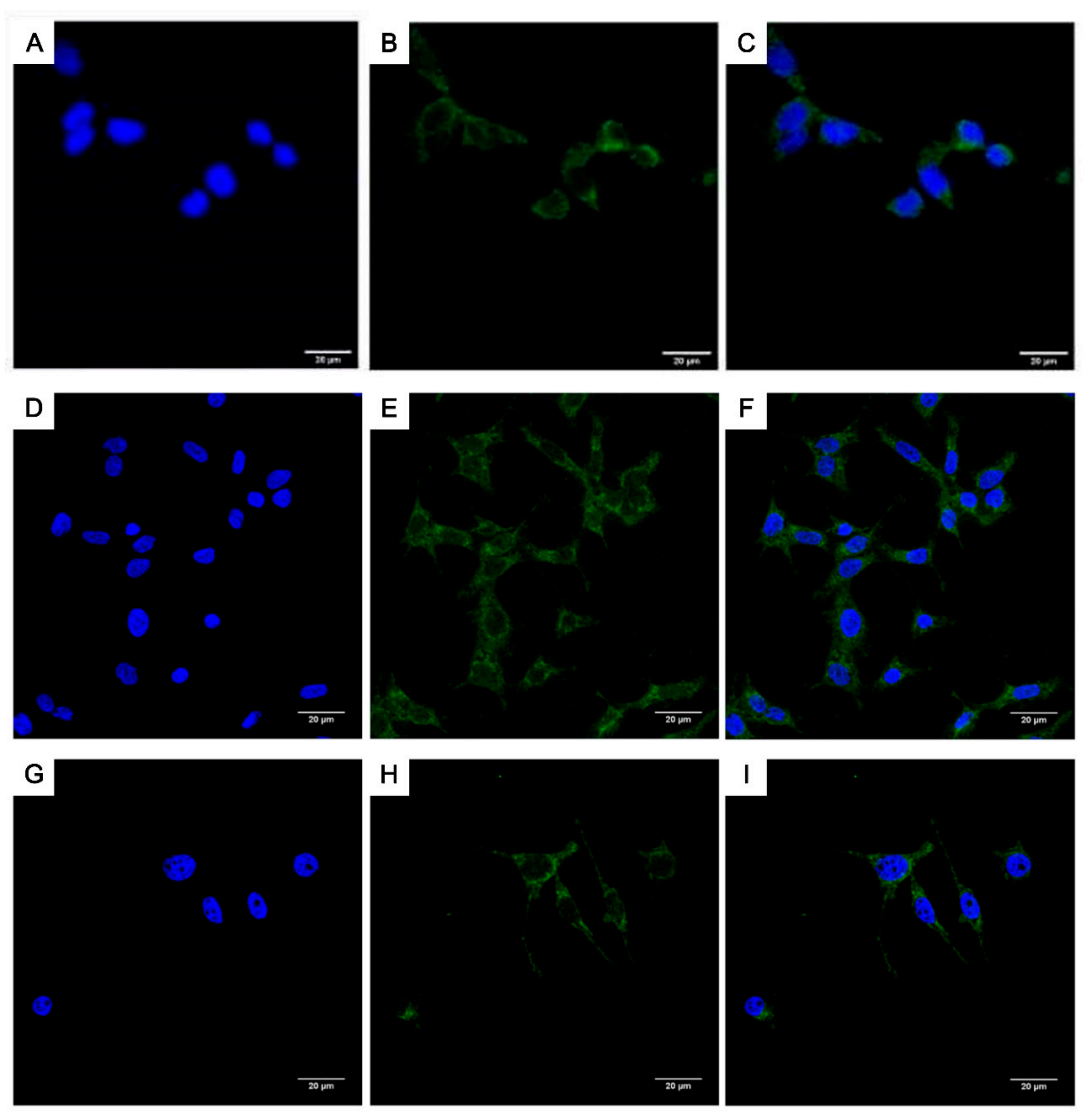
FRs analysis (CLSM) of LoVo cells incubated for 48 h (**A**–**C**) alone (control), and with (**D**–**F**) PEG@MNP or (**G**–**I**) FA-PEG@MNP in the presence of NaBu. In A, D and G nuclei are stained with DAPI (blue); B, E and H green florescence of FRs; in C, F and I a merge was made. Scale bar 20 µm.

In the presence of 10 mM NaBu (Figure 7), similar intensities of the fluorescence signal were observed for cells treated with PEG@MNP and FA-PEG@MNP.

CLSM analysis indicated that without NaBu, FRs were more expressed on the cells treated with FA-PEG@MNP, whereas, in the presence of NaBu no differences were observed between the cells treated with FA-PEG@MNP and PEG@MNP. It was probable that these effects were due to the differentiation induced by NaBu that led to a similar uptake of both MNP types [39]. These results accounted for the similar cell uptake observed for both types of MNPs in the presence of NaBu. These results were confirmed through corrected total cell fluorescence (CTCF) and ANOVA statistical analysis (Figure S7).

The combination of MTT experiments and CLSM analyses indicated that the efficacy of NaBu as an anti-cancer agent is increased by the presence of FA-PEG@MNPs or PEG@MNPs and that, in turn, butyrate affects the internalization process of MNPs.

### 2.4. Cell Morphology: A SEM Study

**Figure 8:**
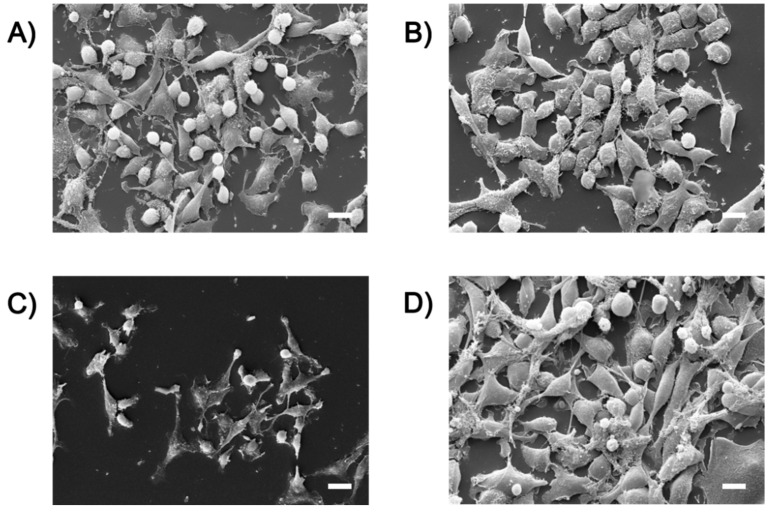
SEM images of (**A**) LoVo cells alone (control) and LoVo cells exposed for 48 h to (**B**) FA, (**C**) NaBu alone and (**D**) NaBu and FA. Scale bar 10 µm.

The combined effects of 1 µg/mL of FA and 10 mM NaBu (both substances used as a free formulations) on cell morphology after 48 h of treatment were studied by SEM (Figure 8). Morphologies of untreated LoVo cells (Figure 8A), of cells exposed to FA (Figure 8B) show similar morphologies. In the presence of NaBu (Figure 8C) clear differences were evident compared to normal morphologies.

In this case, SEM observations indicated a reduced number of cells that were flattened and stretched on the surface with a smooth plasma membrane. Round cells of altered size and forms were also present, probably apoptotic bodies or misshapen cells.

The combined treatment (Figure 8D) of FA and NaBu resulted in the growth of cells with an almost normal morphology (similar to Figure 8A,B), but accompanied by the presence of numerous round cells similar to the ones observed in Figure 8C. These results were coherent with MTT data, since NaBu exposure leads to significant alterations in cell growth, but the presence of FA reduced these alterations acting as a protective agent. The combined effect of NaBu and folate showed that the latter appeared to modulate the cytotoxic action of fatty acid as demonstrated by MTT and morphological SEM analysis. The effect of the combined use of MNPs (15 µg/mL) and NaBu was also studied. For these experiments, two concentrations of NaBu were used: 10 mM and 1 mM.

**Figure 9:**
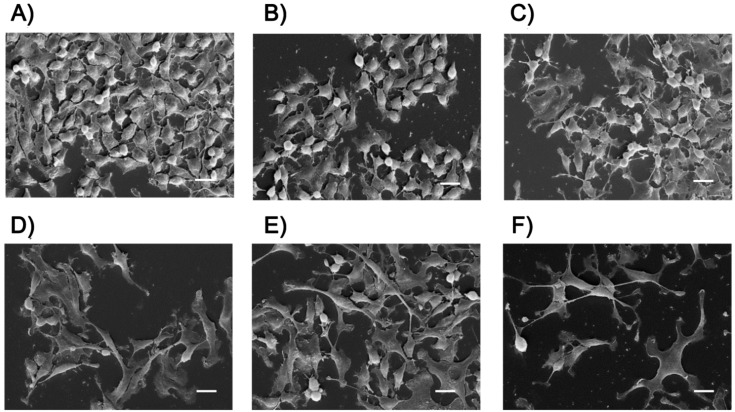
LoVo cells visualized by scanning electron microscopy. Control cells (**A**) in the presence of FA-PEG@MNPs (**B**) and PEG@MNPs (**C**). The cells after NaBu treatment (**D**) and the combined action of FA-PEG@MNPs (**E**) and PEG@MNPs (**F**) with NaBu. Scale bar 10 µm.

At 10 mM NaBu, butyrate-induced differentiation stopped the cell proliferation, thus determining apoptosis and detachment, especially in combination with MNPs. Consequently, SEM samples showed few cells and scattered cellular debris. A typical SEM image of LoVo cells incubated with PEG@MNPs and NaBu (10 mM) is shown in Figure S5. Due to the above-mentioned problems SEM analysis was performed at 1 mM NaBu. Figure 9A shows untreated LoVo cells (after 72 h) grown in vitro forming more or less confluent monolayers. The shape of the cells varied from flattened to rounded depending on their replicative stage. Similar pictures were observed when adenocarcinoma cells were exposed to FA-PEG@MNPs or PEG@MNPs (Figure 9B,C) without the addition of NaBu. Evident effects appeared when cells were exposed to the action of 1 mM NaBu (Figure 9D). Cell morphology was significantly modified as well as their number, and normal round cells, a sign of mitotic activity, were scarcely present. These features were probably due to the differentiating effect of NaBu. The cells were of various shapes and size, flattened on the substrate with cytoplasmic areas that extended in all directions. The plasma membrane was almost devoid of specialization and often seemed to swell to form small blebs. The combined treatment of FA-PEG@MNP and NaBu induced the same alteration as the cellular form, but the cells appeared less flattened, with a more three-dimensional appearance (Figure 9E). Following the simultaneous exposure to PEG@MNPs and NaBu, LoVo cells showed significant changes in shape and size and they appeared more stressed than with butyrate alone (Figure 9F).

The typical differentiation induced by NaBu and, therefore, its cytotoxic action initially provoked the distension and adhesion of the cells on the substrate as well as a reduced proliferation. These characteristics led to the triggering of the apoptotic process and therefore to the loss of cell–cell and cell–substrate contact.

The addition of folic acid seemed to inhibit or reduce the action of butyrate by maintaining a certain degree of proliferation and delaying apoptosis. The combined treatment with NaBu and PEG@MNP showed the greatest cytotoxic effect on LoVo cell morphology (Figure 9F), thus confirming the MTT results shown in Figure 1.

On the other hand, the presence of FA-PEG@MNPs, due to their ability to enter the nucleus, increased proliferation, counterbalancing the effects on transcription due to the presence of NaBu. This behavior resulted in a slightly less cytotoxic effect of the NaBu/FA-PEG@MNPs combination compared to the NaBu/PEG@MNP one.

## 3. Materials and Methods

### 3.1. Materials

Iron(II) chloride tetrahydrate (FeCl_2_·4H_2_O), Iron(III) chloride hexahydrate (FeCl_3_·6H_2_O), ammonium hydroxide (NH_4_OH), ethanol, 3-aminopropylphosphonic acid (NH_2_-PA), methoxypolyethylene glycol acetic acid N-succinimidyl ester (PEG-NHS) with molecular weight (MW) 5000 Da, folic acid (FA), N-hydroxysuccinimide (NHS), N,N-dicyclohexylcarbodiimide (DCC), carboxy-X-rhodamine N-succidinimidyl ester (Rhod-NHS), triethylamine, and dimethyl sulfoxide (DMSO) were purchased from Sigma-Aldrich Chemicals and were used without further purification. Water was of Milli-Q grade (18.2 MΩ cm) and was filtered through a 0.22 µm filter.

### 3.2. Synthesis of Magnetic Fe_3_O_4_ Nanoparticles (MNPs)

Bare iron oxide nanoparticles were synthesized by the alkaline co-precipitation of Fe^3+^ and Fe^2+^ according to the protocol described in the literature [40]. FeCl_2_·4H_2_O and FeCl_3_·6H_2_O (molar ratio 1:2) were dissolved in water (50 mL) under an N_2_ atmosphere with vigorous stirring. NH_4_OH (5 mL, 25%) was added to the solution at 80 °C, and the reaction was continued for 30 min. The resulting suspension was cooled to room temperature and washed with ultrapure water. The obtained bare magnetic nanoparticles (bare MNPs) were isolated from the solvent by magnetic decantation.

### 3.3. Synthesis of N-Hydroxysuccinimide Ester of Folic Acid (FA-NHS)

FA-NHS was prepared by the following published method [41]. FA (500 mg) was dissolved in 10 mL of DMSO with 240 mL of triethylamine. NHS (260 mg) and DCC (470 mg) were added and the mixture was reacted overnight at room temperature in the dark. The by-product, dicyclohexylurea, was removed by filtration. The DMSO solution was then concentrated under reduced pressure and FA-NHS was precipitated in diethyl ether. The product was washed several times with anhydrous ether and air dried.

### 3.4. Synthesis of PA@MNPs

MNPs (200 mg) were dispersed in H_2_O (25 mL) using an ultrasonic bath for 30 min. NH_2_-PA (100 mg) was added and the suspension was agitated for 2 h at room temperature. The particles were separated magnetically and washed four times with H_2_O followed by ethanol and then air dried.

### 3.5. Synthesis of PEG@MNPs

PA@MNPs (300 mg) and PEG-NHS (30 mg) were dispersed in DMSO (15 mL). The solution was mixed overnight at 25 °C. The obtained particles were separated magnetically, washed with DMSO, H_2_O, ethanol and then air dried.

### 3.6. Synthesis of FA-PEG@MNPs

FA-PEG@MNPs were obtained with a similar procedure as described above, but adding FA-NHS to the solution. PA@MNPs (300 mg), PEG-NHS (30 mg) and FA-NHS (3 mg) were dispersed in DMSO (15 mL). The solution was mixed overnight at 25 °C. The obtained particles were separated magnetically, washed with DMSO, H_2_O, ethanol and then dried under air. Carboxy-X-rhodamine marked FA-PEG@MNPs were obtained with the same procedure, but adding Rhod-NHS (3 mg) to the above described solution.

### 3.7. Cell Culture

LoVo cells were cultivated in RPMI 1640, supplemented with Antibiotic-Antimicotic (Fungizon 25 μg/mL, penicillin 10,000 U/mL, streptomycin 10,000 μg/mL), 10% fetal bovine serum (FBS), and maintained in a 37 °C incubator, with humidified atmosphere of 5% CO_2_/95% air. When the cells were approximately 80% confluent, they were detached by Trypsin/EDTA-4Na (0.05%/0.02% w/v) and used for experiments. All products used were purchased from GIBCO (Life Technologies).

### 3.8. Cytotoxicity Assay

LoVo cells were seeded into 24-well tissue culture plates, at a concentration of 3 × 10^4^ in each well and incubated for 24 h. After this pre-adhesion time, the medium was changed with fresh medium containing different MNPs. After 48 and 72 h of incubation, a cytotoxicity assay in the presence of different concentrations of free butyrate, was performed using an MTT test, based on the reduction of yellow 3-(4,5-dimethythiazol-2-yl)-2,5-diphenyl tetrazolium bromide (MTT) by mitochondrial succinate dehydrogenase. The optical density (OD) values at 570 nm of the samples, with background subtraction of OD at 650 nm, were measured using a Cary 50 spectrophotometer (Varian). Cell viability was expressed in % values (ΔOD: 570–650 nm) with respect to the control. All experiments were performed in triplicate.

### 3.9. Sample Characterization: XPS and UV-VIS Spectroscopy

XPS spectra were recorded with a PHI 5600 multi-technique ESCA-Auger spectrometer (Physical Electronics, Chanhassen, MN, USA) with a standard Mg-Kα X-ray source. Analyses were carried out with a photoelectron angle of 45° (relative to the sample surface) with an acceptance angle of ±7°. The XPS binding energy (B.E.) scale was calibrated by centering the C 1s peak due to hydrocarbon moieties and adventitious carbon at 285.0 eV. UV/Vis measurements were carried out with a JASCO V-560 UV/Vis spectrophotometer (JASCO, Easton, MD, USA), and the spectra were recorded with a ± 0.2 nm resolution.

### 3.10. SEM/EDX Microanalysis

After 24 h of pre-adhesion and after 72 h of incubation with the different kinds of nanoparticles, cells were fixed in 2% glutaraldehyde in 0.1 M sodium-cacodylate buffer (EMS), pH 7.2, for 1 h at 4 °C and then post-fixed in 1% osmium tetroxide (EMS) for 1 h at 4 °C. After dehydration in graded ethanol and followed by Critical Point Drying using CO_2_ (Emscope CPD 750), samples were mounted on stubs and sputter coated with gold. Sample morphologies were observed by field emission gun scanning electron microscopes (FE-SEM), using a ZEISS SUPRA VP 55 and a ZEISS EVO LS 10 (ZEISS, Oberkochen, Germany). Chemical analysis was performed by energy dispersive X-ray (EDX) analysis ussing an INCA-Oxford windowless detector with an electron beam energy of 15 keV.

### 3.11. TEM

The cells were initially fixed and post-fixed as above mentioned for SEM analysis; the samples were then scraped, centrifuged (300× *g*/5 min) and the pellets dehydrated in ethanol/acetone and embedded in Durcupan ACM (Fluka). Ultra-thin sections were obtained by an Ultracut Reichert Jung instrument, collected on Cu-Rd grids (EMS) and stained with 5% uranyl acetate and 1% Pb citrate. Finally, the samples were observed and photographed by an S-TEM Hitachi S7000 instrument (Hitachi, Tokyo, Japan).

### 3.12. Confocal Microscopy

To study the intracellular localization of MNPs, Confocal Laser Scanning Microscopy was performed (CLSM, Zeiss LSM700, Jena, Germany). LoVo cells were cultured on glass coverslips and after 24 h of pre-adhesion were incubated with FA-PEG@MNP and PEG@MNP (20 µg/mL) in the presence of NaBu (1 mM). Afterwards the cells were washed three times with phosphate-buffered saline (PBS). Samples were fixed in 4% paraformaldehyde (PFA) in PBS (15 min at room temperature), washed with PBS and mounted with Vectashield containing 4′,6-Diamidino-2-Phenylindole, Dihydrochloride (DAPI) (Vector Laboratories, Inc., Burlingame, CA, USA).

Indirect immunofluorescence to evidence FRs was carried out on LoVo cells treated as above mentioned for 48 h and then fixed in 4% PFA in PBS. After permeabilization, cells were incubated for 1 h with the primary antibody (1/100; FR (E11): sc-515521—Santa Cruz Biotechnology), followed by incubation with the secondary antibody (1/100; m-IgGk BP-FITC sc-516140—Santa Cruz Biotechnology). Slides were mounted with Fluoro Gel—DAPI (EMS) and observed with a Confocal Laser Scanning microscope Leica TCS SP8 (AOBS).

## 4. Conclusions

The present paper investigated the interplay of biocompatible MNPs modified with multifunctional layers containing or not FA and NaBu used as a free formulation.

In the absence of NaBu, MNPs having a coating of pure PEG did not show a significant cellular uptake, whilst the introduction of FA molecules into the organic coating was able to increase cellular uptake by almost ten times as shown by a label free SEM/EDX investigation. In addition, the feature of our system is that, in response to folic acid added either as an MNP coating component or as a free formulation, the amount of FRs in LoVo cells increased on cellular membranes, as demonstrated by confocal microscopy.

In the presence of NaBu, the differences between the two types of MNPs were strongly reduced. We showed that the contemporary exposure of adenocarcinoma cells to NaBu and to MNPs either with or without FA caused a strong decrease of LoVo cell viability, whereas an equal expression of FRs was highlighted. It is likely these effects are due to the differentiation induced by butyrate that leads to a similar uptake of both MNP types [39]. In addition, our findings indicate that folate in the coating or used as a free formulation can modulate the cytotoxic action of NaBu.

Therefore, on the basis of these findings, NaBu treatment when used in combination with magnetic nanoparticles with or without FA, based on specific cancer cell properties, could represent a useful therapeutic tool in future cancer treatment.

## Supplementary Materials

Supplementary materials can be found at https://www.mdpi.com/1422-0067/21/22/8473/s1.
