# Supplementary material for: The Interplay between Fe3O4 Superparamagnetic Nanoparticles, Sodium Butyrate, and Folic Acid for Intracellular Transport
Source: Int J Mol Sci. 2020 Nov 11;21(22):8473. doi: 10.3390/ijms21228473 (PMC7697628; doi:10.3390/ijms21228473)
Supplement: Supplementary file 1 [file ijms-21-08473-s001.pdf]

Supplementary Material

The interplay between Fe_3O_4 superparamagnetic nanoparticles, sodium butyrate and folic acid for the intracellular transport

Maria Teresa Cambria[1,*], Giusy Villaggio[1], Samuele, Laudàni[1], Luca Pulvirenti[2], Concetta Federico[3], Salvatore Saccone[3], Guglielmo Guido Condorelli[2,4,*], Fulvia Sinatra[1]

[1] Dipartimento di Scienze Biomediche e Biotecnologiche, Università di Catania, 95100 Catania, Italy

[2] Dipartimento di Scienze Chimiche, Università di Catania, 95125 Catania, Italy

[3] Dipartimento di Scienze Geologiche, Biologiche e Ambientali, Università di Catania, 95125 Catania, Italy INSTM UdR di Catania, 95125 Catania, Italy

[4] INSTM UdR di Catania, 95125 Catania, Italy

*Correspondence: cambrimt@unict.it (M.T.C.); guido.condorelli@unict.it (G.G.C.)

Details of the material characterization.

Each step of the synthetic route reported in scheme 1 was monitored by XPS. The atomic compositions of bare MNPs, PA@MNPs, PEG@MNPs, FA-PEG@MNPs and FA-PEG@MNPs with rhodamine are reported in Table S1. Negligible P 2p signal and a low N 1s signal due to N-containing adventitious species are present in the bare Fe_2O_3 nanoparticles. After NH_2-PA anchoring the presence of N and P are clear indications of the presence of the NH_2-PA layer. In addition, the N/P atomic ratio close to 1 is consistent with the theoretical N/P ratio of the NH_2-PA molecule. As regard FA-PEG@MNPs and FA-PEG@MNPs with rhodamine, the increased N/P atomic ratio compared to both PA@MNPs and PEG@MNPs is consisting with the anchoring of functional species (FA and rhodamine) containing carbon atoms.
High resolution P 2p, C1s and N 1s XPS region give further indications of the success of the functionalization steps.
After the anchoring process of NH_2-PA, the P 2p band at 133.2 eV was observed (Figure S1a left), thus indicating the presence of the phosphonic acid on the surface. In addition, the B.E. position is shifted towards lower values compared to those typical for phosphonic acid, suggesting that both terminal –POH groups are deprotonated due to the occurrence of two P–O–Fe bonds [40,42 and reference therein]
The P 2p band does not change after the anchoring of the other functional molecules (Figure S1 spectra b-d, left), thus indicating that the phosphonic acid is not removed during the immobilization of the functional molecules.
The N 1s band of PA@MNPs (Figure 2a, right) consists of two components of comparable intensity. The component at 399.9 eV is associated with the primary amine (–NH_2) groups of the anchored aminopropylphosphate, whilst the component centered at 401.5 eV is due to the amino groups interacting with the Fe_3O_4 surface through protonation or formation of –H bonds [42]. Some changes of the N1s shape (Figure 2, spectra b-d right) have been observed after the grafting of functional molecules PEG, FA and Rhodamine. In particular, the component at about 399.9 eV increases due to the formation of amide bonds (400.2 eV) [40] between the functional molecules and PA-NH_2. Note that the intensity enhancement of this component is more evident for FA-PEG@MNPs and FA-

PEG@MNPs with rhodamine compared to PEG @MNPs, due to the presence of several nitrogen atoms in FA (B.E. ranging from 400 to 399 eV) [40 and references therein].

The C 1s XPS regions of the different coatings namely PA@MNP, PEG @MNP, FA-PEG @MNP and FA-PEG @MNP with rhodamine are reported in Figure S1 a-d, middle.

The C 1s band of PA@MNPs (Figure S1a) consists of a single peak at 285.0 eV, assigned to aliphatic carbons. The spectra of PEG@MNPs, FA-PEG@MNPs and FA-PEG@MNPs with rhodamine (Figure S1 b-d) show, beside the main peak at 285.0 eV due to aliphatic and aromatic carbons, an enlargement towards higher B.E. due to a component around 286 eV arising from the oxygen-bonded carbons of PEG. In addition, in the case of FA-PEG@MNPs and FA-PEG@MNPs with rhodamine, a component around 288.6 eV due to carboxylic and amidic groups of FA and Rhodamine can be observed.

The anchoring of FA on the functionalized MNPs has been also proved by UV-Vis spectra of a FA-PEG@MNPs and FA-PEG@MNPs and FA-PEG@MNPs with rhodamine colloidal solutions. Figure S2 compares the spectra of PEG@MNPs, FA-PEG@MNPs and FA-PEG@MNPs with rhodamine colloidal dispersions. The spectrum of a FA solution (2 μM) has been added as reference. An evident band at 274 nm typical of the FA is clearly visible in the reference and in the FA-PEG@MNPs and FA-PEG@MNPs with rhodamine colloidal solutions, thus confirming the presence of FA in the MNPs.

	**MNPs**	**PA@MNPs**	**PEG@MNPs**	**FA-PEG@MNPs**	**FA-PEG@MNPs with Rhod.**
Fe $2p_{3/2}$	17.2	16.2	16.1	13.2	12.1
O 1s	69.3	46.2	40.8	41.4	39.8
C 1s	12.9	31.1	37.3	38.9	42.2
P 2p	noise level (~0.1)	3.1	2.4	2.0	1.5
N 1s	0.5	3.0	2.1	2.6	2.9

TABLE S1: XPS estimated surface atomic concentrations of a) PA@MNPs, b) PEG@MNPs, c) FA-PEG@MNPs and d) FA-PEG @MNPs with Rhodamine.

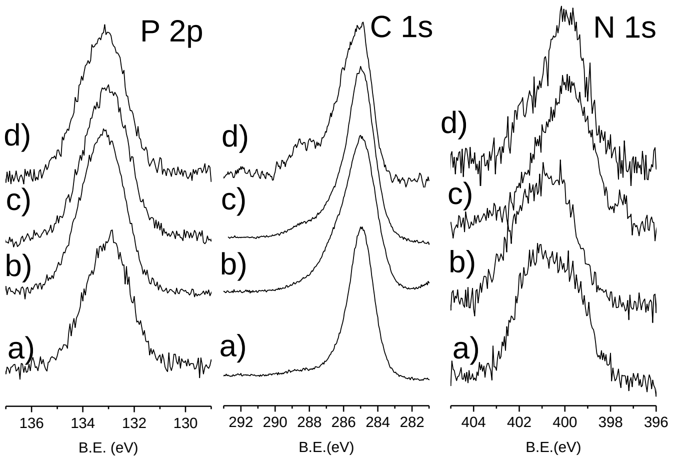

Figure S1: High resolution P 2p, C 1s and N 1s spectral regions of a) PA@MNPs, b) PEG@MNPs, c) FA-PEG@MNPs and d) FA-PEG@MNPs with Rhodamine.

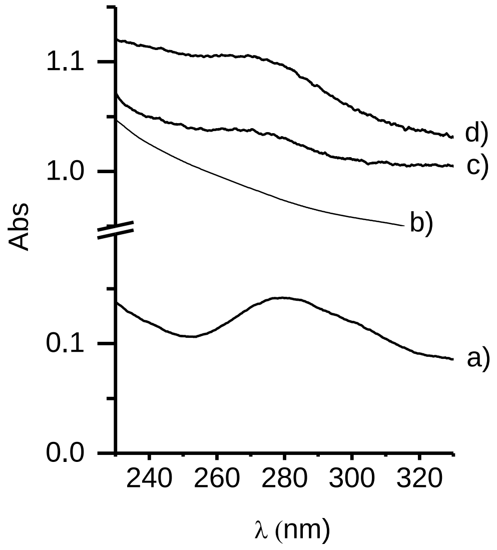

Figure S2: UV/Vis spectra of a) FA-NHS solution (2 μM) and b) PEG@MNPs, c) FA-PEG@MNPs with Rhodamine and d) FA-PEG@MNPs colloidal dispersions.

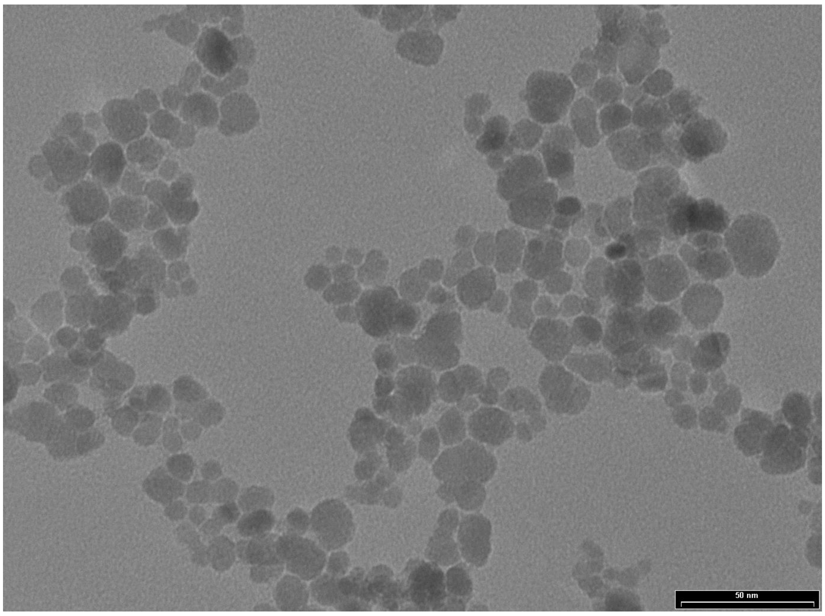

Figure S3. TEM of FA-PEG@MNPs. Similar image was obtained for PEG@MNPs.

Fe/Au ATOMIC RATIO		
FA-PEG@MNPs	PEG@MNPs	Control
1.91	0.21	0.04

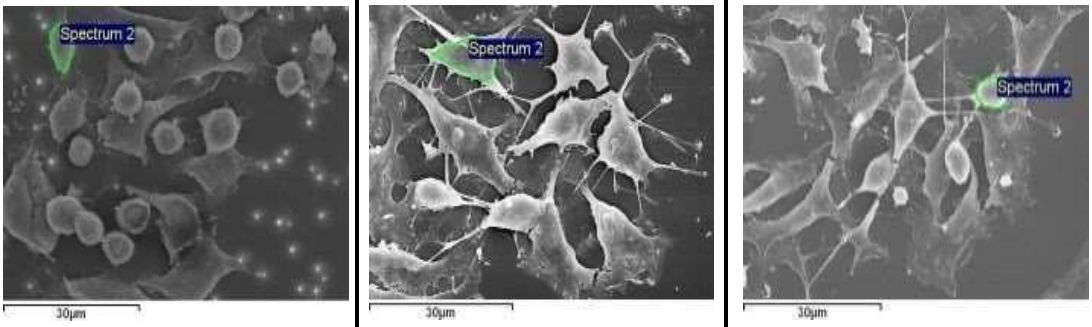

Figure S4. Average Fe/Au values of control and MNPs-treated samples obtained from EDX analyses performed in correspondence of the cells. Examples of the typical region analyzed for determining the Fe/Au ratio in a cell are reported below.

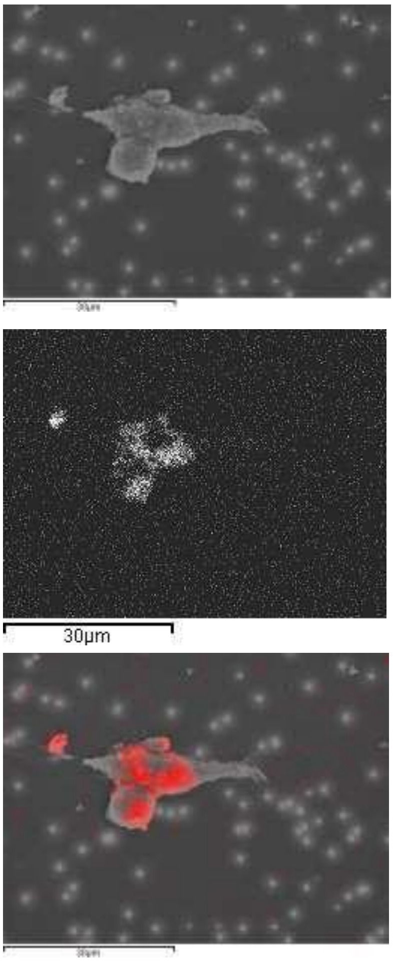

Figure S5. a) typical SEM images, b) EDX maps of Fe distribution (bright spots) and c) overlay images of SEM and EDX Fe distribution (Fe spots colored in red) of samples of cells incubate with PEG@MNPs and NaBu (10mM). Similar images were observed for cells incubate with FA-PEG @MNPs and NaBu (10mM).

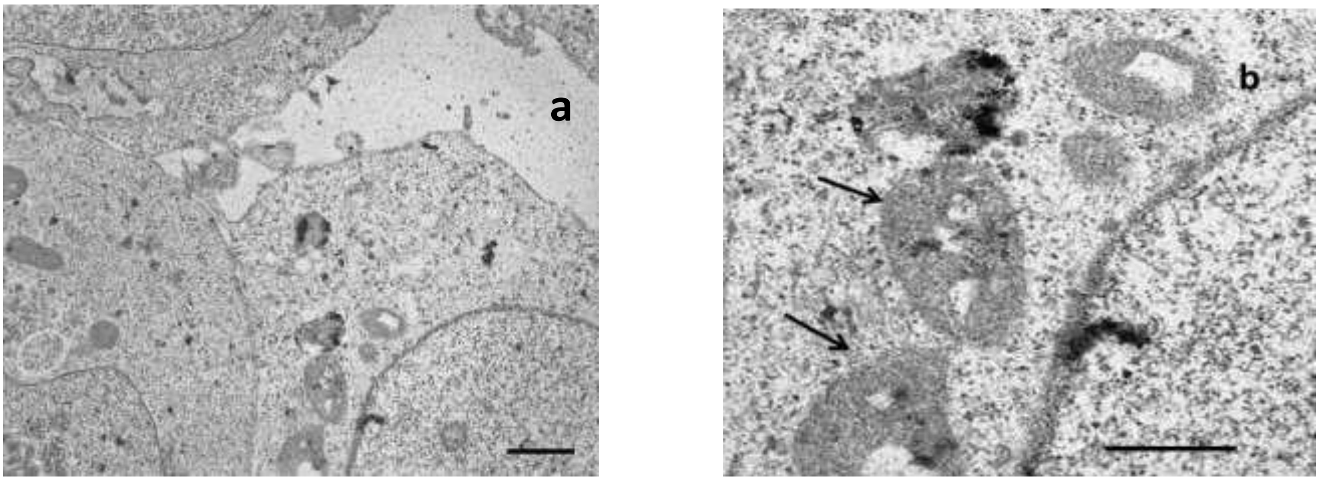

Figure S6. TEM Images of uptake of FA-PEG@MNP after 72 h in Lovo cells (a) and in b) mitochondria (black arrows) with enlarged christae and aggregates of MNPs in the matrix. Scale bar: a = 1 µm, b = 0.5 µm

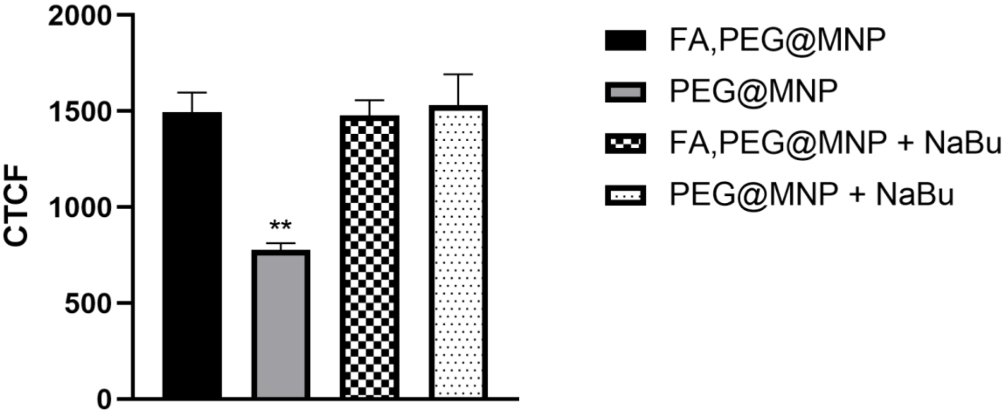

Figure S7. CTCFs relative to Figures 7 and 8. Statistical analysis One-way ANOVA (Tukey's multiple comparison correction) was performed using GraphPad prism 8. **$P \leq 0.01$ PEG@MNP vs other groups
